# A Study on the Phenotypic Variation of 103 Cucumber (*Cucumis sativus* L.) Landraces for the Development of Desirable Cultivars Suitable for the Changing Climate

**DOI:** 10.3390/life12081235

**Published:** 2022-08-15

**Authors:** Iftekhar Ahmed, Md. Motiar Rohman, Md. Amir Hossain, Md. Rezwan Molla, Md. Golam Azam, Md. Mahadi Hasan, Ahmed Gaber, Bander Albogami, Akbar Hossain

**Affiliations:** 1 Plant Genetic Resources Centre, Bangladesh Agricultural Research Institute, Gazipur 1701, Bangladesh; 2 Department of Genetics and Plant Breeding, Bangladesh Agricultural University, Mymensingh 2202, Bangladesh; 3 Plant Breeding Division, Bangladesh Agricultural Research Institute, Gazipur 1701, Bangladesh; 4 Regional Agricultural Research Station, Bangladesh Agricultural Research Institute, Pabna 6620, Bangladesh; 5 State Key Laboratory of Grassland Agro-Ecosystems, College of Ecology, Lanzhou University, Lanzhou 730050, China; 6 Department of Biology, College of Science, Taif University, P.O. Box 11099, Taif 21944, Saudi Arabia; 7 Department of Agronomy, Bangladesh Wheat and Maize Research Institute, Dinajpur 5200, Bangladesh

**Keywords:** cucumber, characterisation, genetic variability, climate change, food security

## Abstract

The cucumber (*Cucumis sativus* L.) is one of the most important vegetables in Bangladesh as well as across the globe. However, many of the important cucumber landraces have disappeared in Bangladesh due to climate change, particularly erratic rainfall, extreme temperature, salinity, and drought. Therefore, to protect against the extinction of the cucumber landraces, we collected 103 landraces in different geographical regions of Bangladesh, including drought and saline-prone areas, and studied their divergence for the future breeding programme for the development of cultivars suitable for the climate-changing situations. Data on morphological features, yield, and its components, which include 17 qualitative and quantitative traits, were recorded during the observation. Among the cucumber landraces, the Shannon–Weaver diversity index analysis revealed the presence of genetic diversity in these landraces. The biggest diversity appeared in the fruit-related characteristics, i.e., stem end fruit shape, bottom end fruit shape, fruit shape, and fruit skin colour at the table and harvest maturity. The descriptive statistics and analysis of variance expressed a wide range of variability for quantitative traits. A broad phenotypic variation was also observed for traits such as yield plant^−1^ [CV (%) 31.88, ranges 0.96 to 3.11 kg] and fruits plant^−1^ (CV (%), 28.71, ranges, 2.58 to 9.75). High heritability (broad sense) coupled with a high genetic gain was observed for yield and yield-contributing characteristics, indicating that these characteristics are controlled by additive gene effects, and they are more reliable for effective selection. The phenotypic correlation studies showed that fruit yield plant^−1^ exhibited a positive and significant correlation with fruits plant^−1^, fruit length, fruit weight, fruit width, branches plant^−1^, and plant height. All landraces were grouped into six clusters, and the maximum number of landraces were accommodated in cluster VI (30), followed by cluster V (22), cluster III (22), cluster IV (14), cluster I (13), and cluster II (2). Comparing cluster means with studied traits revealed that cluster III with landraces AC-14, AC-97, AC-471, AC-451, and RAI-209 were more divergent for improving average fruit weight, fruit length, and fruit width. On the other hand, cluster IV with landraces AC-201, TT-161, RAI- 217, RAI-215, and TRMR-103 were more divergent for improving average vine length, internode length, and the number of primary branches plant^−1^, the number of fruits plant^−1^, and yield plant^−1^. According to the MGIDI index, AC-14 (G1), AC-201 (G7), AC-471 (G24), AC-97 (G30), RAI-215 (G68) and TT-161 (G 94) may be considered to be the best parents based on their qualitative and quantitative characteristics for the future breeding programme. Moreover, crossing between the landraces, which were collected from saline and drought areas, in clusters I, V, and VI with those in other clusters could produce suitable cucumber varieties for the climatic changing situation.

## 1. Introduction

The economy of Bangladesh is primarily agrarian, where the agricultural sector contributes to about 16% of GDP and to more than 60% of employment. However, due to its geographical position, the country is highly vulnerable to climate change, resulting in erratic rainfall, extreme temperatures, increasing levels of salinity, and droughts [1]. It has been projected that the mean temperature of Bangladesh will be increased by about 1.0, 1.4, and 2.4 °C by the years 2030, 2050, and 2100, respectively [2]. These variations in temperature could lead to reducing crop productivity by up to 30%, creating a very high risk of hunger [3]. The dilemma becomes even bigger because fluctuations in annual rainfall and temperature negatively affect crop growth and encourage attacks by crop pathogens [4]. It has been reported that there are 10–25% reductions in the yields of major staple crops, including wheat, maize, and rice, due to per degree rises in temperature [5]. This is due to an increase in temperature accelerating the metabolic activities of insects and enhancing insects’ food consumption rate. Likewise, the elevated level of CO_2_ makes soybeans more susceptible to insect pathogens [6]. Similarly, for vegetables which are highly vulnerable to the changing climate, the cucumber (*Cucumis sativus* L.) is the most important one. According to Rashid et al. [7], there are 13% yield declines with per unit increases in salinity in cucumbers. In fact, such situations over the long term can degrade lands and resources and affect biodiversity and agricultural production. Genetic diversity is the key pillar of biodiversity and diversity within species, between species, and for ecosystems (CBD, Article 2). Diversity in plant genetic resources (PGRs) provides the opportunity for plant breeders to develop new and improved cultivars with desirable characteristics, which include both farmer-preferred traits (yield potential, large seeds, etc.) and breeders’ preferred traits (stress tolerance, pest and disease resistance, photosensitivity, etc.). Climate change can also affect the ability of many crops’ wild relatives, which are potential gene donors for crop improvement programmes, to survive in their current locations. It has been estimated that between 16 and 22% of plant biodiversity, including the wild relatives of many crop species, may be in danger of extinction by 2055 due to climate change [8]. According to the *Encyclopedia of Flora and Fauna of Bangladesh*, 486 vascular plants are threatened in Bangladesh [9]. Therefore, various initiatives should be undertaken toward the conservation of crop biodiversity.

The cucumber is a member of the Cucurbitaceae family, which comprises 90 genera and 750 species [10]. The genus *Cucumis* includes 32 annual and perennial species divided into two very distinct groups defined by geographic origin and chromosome number (African 2*n* = 24 and Asiatic group 2*n* = 14.) [11]. The African group includes the melon (*C. melo* L.), and the Asiatic group includes the cucumber (*C. sativus*) and its probable ancestor *C. sativus* var. *hardwickii* [12]. The cucumber has been widely consumed all over the world, either fresh or in processed forms such as a sliced or pickled mature fruit after cooking. Wide variation within the species has been observed concerning bearing habits, maturity, yield, shape, size, colour, spines, vine habits, etc.

A huge number of landraces with a wide range of variability in size, shape, and colour of cucumber fruits are still available in Bangladesh. A total of 217 cucumber landraces were collected from different parts of Bangladesh, including stress-prone areas of Bangladesh. Mostly two distinct fruit morph-types are found in Bangladesh: one is the round fruited type called ‘Khira’, and the other is a long fruited type called ‘Shosha’ [13]. Khira is available in late winter, and Shosha is grown throughout the year. Some of the popular cucumber cultivars are location-specific, such as the ‘Potia giant’ in Chittagong, ‘Morma’ in Barishal, ‘Marpha’ in hill districts, and ‘Naogoan khira’ and ‘Vui sosha’, which, in Jessore, are the most popular cultivars grown in Bangladesh. Some areas of Bangladesh (Jessore, Dhaka, Rangpur, and Chittagong regions) are famous for commercial cucumber cultivation. This growth has been tremendous and has contributed to changes in existing farming practices that are replacing traditional landraces of the crop. The yield potential of traditional cucumber cultivars in Bangladesh is 15 t ha^−1^ [8,14], which is much lower than global commercial varieties. As a result, the landraces, possessing valuable biotic and abiotic tolerance genes, are at a high risk of extinction. However, they may contain important genes associated with biotic and abiotic stress tolerance. In this aspect, it is essential to collect the available landraces of the cucumber to stop the depletion of its valuable gene pool as well as to use them in developing varieties worthy of combating climate change situations.

To meet the challenges in crop improvement, efforts were made to widen the genetic base by collecting and conserving landraces across the country. The success of any breeding programme depends much on the genetic diversity of the population available to the breeders as a source of obtaining transgressive segregates with desirable combinations and the judicious selection of parents [15,16]. Genetic markers (heritable characters) are associated with economically important traits and can be used by plant breeders as selection tools [12,17]. Genetic diversity between individuals and populations can be determined using morphological and molecular markers, and morphological representation is the chief step in the explanation and understanding of genetic means [18]. Different attributes in qualitative and quantitative characters are important objects of improvement in fruits and vegetables because of their direct influence on the commercial value of the product. Hierarchical cluster analysis highlights the nature of relationships between and among the types of samples as described by descriptors. It classifies the landraces into different groups based on Euclidian distance and chooses parental lines that can yield superior hybrids [14]. Principal component analysis (PCA) usually suggests the traits that contribute a lot, a little, or not at all to the variation among treatments. Cluster analysis and PCA are tools that can estimate the genetic variability and heritability of a particular set of landraces of a crop, and the multi-trait genotype–ideotype distance index (MGIDI) allows a more efficient and accurate treatment recommendation based on desired or undesired characteristics. A selection index based on these statistical tools for selecting landraces of multiple traits and/or recommending treatments has been proposed by Olivoto and Nardino [19].

Therefore, the current study was undertaken for understanding the phenotypic-based genotypic variation of 103 landraces using those statistical useful tools to find distinct variable groups among the landraces studied. Thus, this study can help in the identification of desirable diverged parents to formulate a new future breeding programme for the development of new cultivars suitable for multiple environments of Bangladesh, particularly for drought and saline-prone areas, in the climate-changing situation.

## 2. Material and Methods

### 2.1. Experimental Site

The experiment was conducted at the experimental field of the Plant Genetic Resources Center (PGRC) of the Bangladesh Agricultural Research Institute (BARI), Gazipur, Bangladesh, during Kharif-1, 2018 at 23°98′ latitude, 90°41′ longitude. The soil of the experimental field was silty clay with a pH of 6.

### 2.2. Experimental Materials, Methods, and Other Operations

A total of 100 locally collected landraces from different areas of Bangladesh, along with 3 commercially released varieties from private companies, were used in this experiment (Table 1).

Landraces were collected from saline-prone (Khulna, Satkhira, and Patuakhali districts) and drought-prone areas (Rangpur, Rajshahi, Kurigram, and Dinajpur districts) of Bangladesh. All 100 landraces were arranged in an augmented design (augmented RCBD) with three check varieties, and four blocks were followed in this study. All check varieties received four replications, giving a total of 112 experimental plots. The plot size was 2 m × 2 m. Each genotype was planted in one plot containing two pits with four plants. The spacing from pit to pit was 1 m, and from plot to plot it was 1 m. Seedings were produced in a seedling plot on 2 March 2018. Twenty-four-days-old seedlings were transplanted into the organised pits of the prepared experimental plot on 26 March 2018. Fertiliser doses were 5 tons ha^−1^ cow dung, 173 kg ha^−1^ urea, 160 kg ha^−1^ triple superphosphate (TSP), 128 kg ha^−1^ muriate of Potash (MoP), gypsum 150 kg ha^−1^, and 8 kg ha^−1^ borax [20]. All of the organic manure, TSP, gypsum, and one-third of urea and MoP were applied during the final land preparation about one week before transplanting. Urea and MoP were supplied in the three equal splits. The trail was prepared just after one week of transplanting, and weeding was performed at one-week intervals. Irrigation was carried out after prolonged drought conditions to ensure the optimal growth of the plant. Pollination was controlled by covering female flowers after selfing with butter paper. Propiconazole at 2 mL/L, Mancozeb at 2 mL/L, and Carbaryl at 1 mL/L of water were sprayed for controlling diseases and insects. A sex pheromone trap was used to control fruit flies.

A total of 17 qualitative traits and 17 quantitative traits (Table 2) were considered during the morphological characterisation based on minimal descriptors for cucurbits [21] descriptors and the guidelines for the conduct of the test for distinctness, uniformity, and stability for cucumbers [22].

### 2.3. Statistical Analysis

The phenotypic diversity for qualitative traits was determined by using the Shannon–Weaver diversity index (SWDI) [23], as defined by Jain et al. [24]. Statistical analyses were performed under the R-statistics platform (software version 4.0.2) [25]. Analyses of variance (ANOVA) for each trait were assessed by using the R package ‘augmented RCBD’ [26].

#### 2.3.1. Estimates of Variability Parameter

Variability estimates, including genotypic and phenotypic variances, heritability, genotypic and phenotypic coefficients of variations, and the genetic advance were estimated according to [27,28,29,30].

#### 2.3.2. Phenotypic and Genotypic Variance

These parameters were calculated according to the formula given by [30] for genotypic variance:

$$\sigma^{2}g=\frac{\mathrm{MSG}\;-\;\mathrm{MSE}}{r}\;\times \;100$$

where MSG is the mean sum of the square for the landraces; MSE is the mean sum of the square for the error; and r is the number of replications.

#### Phenotypic Variance

The phenotypic variance was calculated as follows:

σ^2^p = σ^2^g + σ^2^e

where σ^2^g is the genotypic variance, and σ^2^e is the environmental variance equal to the error mean square.

#### 2.3.3. Genotypic and Phenotypic Coefficients of Variation

The genotypic and phenotypic coefficients of variation were calculated with the following formula [31]:

$$\mathrm{GCV}=\frac{\sigma g\;\times 100}{\bar{X}}$$

$$\mathrm{PCV}=\frac{\sigma p\;\times 100}{\bar{X}}$$

where GCV is the genotypic coefficient of variation; PCV is the phenotypic coefficient of variation; σ g is the genotypic standard deviation; σ p is the phenotypic standard deviation; and $\bar{X}$ is the population.

#### 2.3.4. Estimation of Heritability

Heritability, in a broad sense for all the traits, was computed as suggested by [21]:

$$h^{2}(\%)=\frac{\sigma^{2}g\;}{\sigma^{2}p}\;\times \;100$$

where h^2^ is heritability in a broad sense; σ^2^g is the genotypic variance; and σ^2^p is the phenotypic variance. The heritability was classified as low (0–30%), moderate (30–60%), or high (>60%), as suggested by [32].

#### 2.3.5. Estimation of Genetic Advance

The genetic advance was calculated as follows:

$$\mathrm{GA}=h^{2}\;\cdot \;b\;\cdot \;K\;\cdot \;\sigma p\;\mathrm{or}\;\mathrm{as}\;\mathrm{genetic}\;\mathrm{advance}:\;\mathrm{GA}=K\;\cdot \;\frac{\sigma^{2}g}{\sigma^{2}p}\;\;\cdot \;\sigma \mathrm{ph}$$

where K is the selection intensity or the value that is 2.06 at a 5% selection intensity; σp is the phenotypic standard deviation; h^2^ is heritability in a broad sense; σ^2^g is the genotypic variance; and σ^2^p is the phenotypic variance.

Mathematical figures were plotted using the ggplot2 package [33]. The hierarchical clustering was performed using the Euclidean distance matrix. Principal component analysis (PCA) was performed using R package ggplot2. A G x T biplot was constructed by a two-way matrix for 17 traits and 103 landraces (100 landraces + 3 checks). The landraces were plotted according to scores on each principal component, and similarly, traits were plotted based on the eigenvectors on each principal component. The R package ggplot2, scales, and GGally were used for the heatmap analysis. The MGIDI index was used to rank the treatments based on the desired values of the studied traits. The MGIDI [19] was computed as follows:

$$\mathrm{MGIDIi}={\left[\overset{f}{\underset{j=1}{\sum }}{\left(\gamma_{\mathrm{ij}}-\gamma_{j}\right)}^{2}\right]}^{0.5}$$

where γij is the score of the ith genotype in the j^th^ factor (i = 1, 2,…, t; j = 1,2,…, f), with t and f being the number of landraces and factors, respectively, and γj is the jth score of the ideal genotype. The genotype with the lowest MGIDI is then closer to the ideal genotype and thus indicates the desired values for all the measured traits. The selection differential for all traits was performed considering a selection intensity of ~20%. The index computation was performed in the R software using the ‘metan’ package [19].

## 3. Results

### 3.1. Qualitative Traits

The morphological observations of 103 landraces using 17 descriptors are presented in Table 3. The observed traits had varied stem characteristics, leaf characteristics, fruit characteristics and seed characteristics. The same plant growth type was shown in all the plants observed, and all of them were indeterminate. Plant growth was viny amongst 91.26% of the genotype, and the rest was intermediate. For the stem characteristics, all of the accessions had different degrees of green colour. Most of the landraces comprised a green coloured stem (70.87%), followed by a dark green (25.24%) and a light green (3.88%) stem. Dense pubescence on the stems appeared in most of the landraces (59.22%), and the rest were intermediate (40.78%). Light green (51.46% of the genotype), green (39.08%), and dark green (8.74%) coloured leaves were found among the studied genotype, and all of them were orbicular-shaped.

Dense pubescence was also observed on leaves and on stems in all accessions, and leaf pubescence was softer than that on the stems. The terminal leaf apex shape appeared obtuse in the maximum genotype (69.9), whereas a rounded shape was in the minimum genotype (30.1). For flower characteristics, the monoecious sex type with the yellow flower was common among the cucumber landraces. The biggest diversity appeared in the fruit-related characteristics. The stem end fruit shape was categorised into necked, acute, and obtuse, and the obtuse stem end fruit shape was the highest (69.9%), followed by the acute (29.13%) and necked (0.97%) stem end fruit shape. The flat blossom end of the fruit was observed among the majority of the genotype (97.08%), but the rest was deep raised. Fruit skin textures were exhibited as smooth (30.09) and wrinkled (69.90). Fruit shapes were found as oblong (84.47%), oval (4.85%), ellipsoid (3.88%), and ovate (6.80%). Fruit skin colour at the table maturity stage was also exhibited in four categories, viz., light green (65.05%), green (14.56%), dark green (3.88%), yellowish green (12.62), greenish yellow (1.94%), blackish green (0.97), and whitish-green (0.97%). A brown fruit skin colour at maturity was recorded in the maximum genotype (69.9%), whereas a yellow colour was presented in the minimum number genotype (30.1%). Seeds’ colours were exhibited as white (41.75%) and cream (58.25%) colours.

Diversity indices are used to summarise the diversity of a population, in which each member belongs to a unique group. The Shannon Diversity Index (sometimes called the Shannon–Wiener Index) is a way to measure the diversity of a species in a community. This normalises the Shannon diversity index to a value between 0 and 1. Note that lower values indicate more diversity, whereas higher values indicate less diversity. According to the table, seed colour, fruit skin texture, and leaf intensity of green colour exhibited a high Shannon–Wiener Index value, which indicates the richness of the genotype among the population for those characteristics. Therefore, the collection should be performed under the consideration of those characteristics and whether the indices are moderate to low.

### 3.2. Analysis of Variance

In the experiment, 103 cucumber landraces were tested for 17 quantitative traits. The analysis of variance revealed significant differences among the landraces for most of the traits studied (Table 4). Out of 17 traits, 13 traits exhibited highly significant results for characteristics, namely: vine length, the number of branches per plant, internode length, days to a male flower, leaf length, leaf width, days to harvest, fruit length, fruit width, fruit weight, the number of fruits per plant, yield per plant, and hundred-seed weight. There were no significant differences in 4 characteristics, namely: days to a female flower, the number of nodes to a male flower, the number of nodes to a female flower, and days to the first fruit harvest. A similar trend was found regarding check and accession by check interaction. The adjusted blocks were insignificant for all the traits. Significant differences indicated the presence of a good deal of variability with the respect to variation in quantitative traits.

### 3.3. Descriptive Statistics

The data on variability parameters, including means, standard deviations (STD), minimums (Min), maximums (Max), and coefficients of variation, are summarised in Table 5. All the quantitative characteristics showed a wide range of variability. The vine lengths ranged from 159.34 cm to 299.61 cm. A wide range was observed for the node number bearing the first female flower (6.21–13.37) and for days to marketable maturity (53.17–71.17), which determine the earliness of a genotype (Table 5).

**Figure 1:**
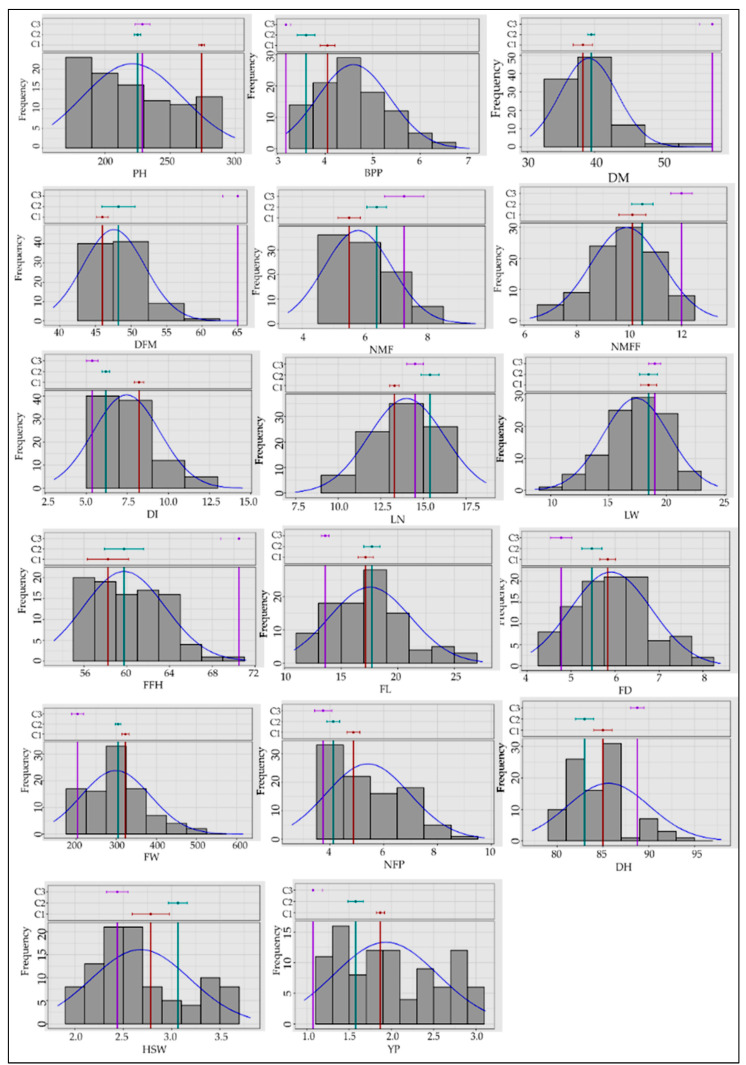
Frequency distribution of the landraces based on quantitative traits: PH = Vine length, BPP = Number of primary branches per plant, DI = Internode distance/internode length, DM = Days to a male flower, DFM = Days to a female flower, NMF = Number of nodes at the first appearance of a male flower, NMFF = Number of nodes at the first appearance of a female flower, LN = Leaf length, LW = Leaf width, FFH = First fruit harvest, FL= Fruit length, FD = Fruit width, FW = Fruit weight, NFP = Number of fruits per plant, DH = Days to mature fruit harvest, YP = Yield per plant, and HSW = Hundred-seed weight.

The number of branches per plant is one of the important yield-contributing characteristics to containing female flowers from lateral branches, which ranged from 3.17 to 7.04. Fruit length, fruit width, and fruit weight are also major yield-contributing traits. Wide variations were observed concerning fruit length (10.80–27.49 cm), fruit width (4.08–8.40 cm), and fruit weight (143.06–615.89 g). Remarkable variations in the number of marketable fruits per plant (2.58–9.75) and yield per plant (0.96–3.11 kg) were obtained. The coefficient of variation was lowest for days to a mature fruit harvest (5.23%), followed by the first fruit harvest (6.04%). However, the highest coefficient of variation value was recorded for yield per plant (31.88%), followed by the number of fruits per plant (28.71%) and fruit weight (28.86%) (Figure 1).

### 3.4. Genetic Variability Component

The results of genetic parameters, viz., the phenotypic coefficient of variation (PCV), genotypic coefficient of variation (GCV), broad-sense heritability (h^2^ BS), and genetic advance as the per cent of the mean (GAM), for all the 17 traits are summarised in Table 6. The highest GCV and PCV were recorded in yield per plant (30.73 and 32.41), followed by fruit weight (29.1 and 30.02) and the number of fruits per plant (26.57 and 28.84), respectively. Genotypic and phenotypic coefficients of variation were found to be high for internode distance, individual fruit weight, and the number of fruits plant^−1^ and yield plant^−1^, indicating a wide range of variation and offering better scope for improvement through selection. However, fruit length showed a medium genotypic and high phenotypic coefficient. Medium/moderate GCV and PCV were found in vine length, the number of primary branches per plant, leaf length, leaf width, fruit width, and hundred-seed weight, showing more influence of the environment on these characteristics.

Hence, selection is not effective in such a case. Low GCV and PCV were found in days to the first male and female flower, the number of nodes at the first male and female flower, days to the first fruit harvest, and days to a mature fruit harvest. The characteristics such as vine length, the number of primary branches per plant, internode distance, leaf length, leaf width, fruit length, fruit width, fruit weight, number of fruits per plant, hundred-seed weight, and yield per plant showed high genetic variability coupled with a high genetic advance, which indicates that these characteristics were under additive gene effects, and hence these characteristics are more reliable for effective selection. Hence, selection based on these characteristics is more useful for the improvement of this crop towards higher fruit yields and quality production.

### 3.5. Analysis of the Correlation Matrix

**Figure 2:**
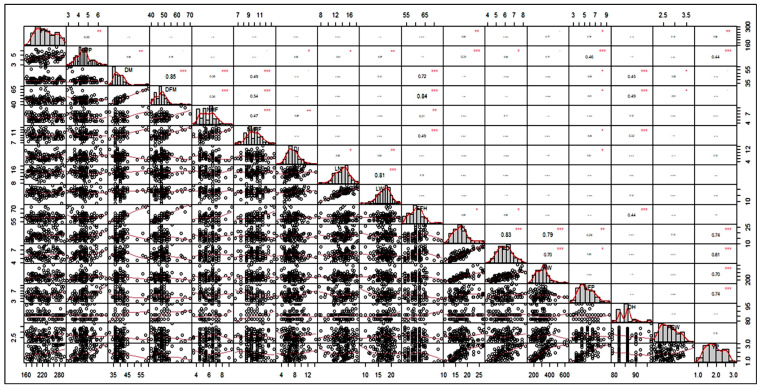
Correlation matrix, scatter plot, and phenotypic frequency distribution of traits; * *p* < = 0.05; ** *p* < = 0.01; *** *p* > 0.001; PH = Vine length, BPP = number of primary branches plant, DI= Internode distance/internode length, DM= Days to a male flower, DFM = Days to a female flower, NMF = Number of nodes at the first appearance of a male flower, NMFF = Number of nodes at the first appearance of a female flower, LN = Leaf length, LW = Leaf width, FFH = First fruit harvest, FL = Fruit length, FD = Fruit width, FW = Fruit weight, NFP = Number of fruits per plant, DH = Days to mature fruit harvest, YP = Yield per plant, and HSW= Hundred-seed weight.

The phenotypic correlation analysis was used to explore linear relationships between various traits, which are visualised in the correlation matrix (Figure 2). The phenotypic correlation studies showed that fruit yield plant^−1^ exhibited a positive and significant correlation with fruits plant^−1^ (0.74 ***), fruit length (0.74 ***), fruit weight (0.70 ***), fruit width (0.61 ***), branches plant^−1^ (0.46 ***), and plant height (0.30 **), which indicate the importance of these traits in the selection for yield. Vine length showed a positive correlation with branches plant^−1^, fruit length, fruit weight, and fruits plant^−1^. Direct selection based on these traits would result in the simultaneous improvement of the aforementioned traits and fruit yield plant^−1^ in cucumbers.

### 3.6. Multivariate Analysis

Multivariate techniques are proven tools used for the estimation of variability and relationships among accessions. To understand the relationship among 103 cucumber landraces with various morphological quantitative traits, principal component and heatmap analysis were conducted.

#### 3.6.1. Principal Component Analysis

The result of PCA for the quantitative traits of cucumber landraces is presented in Table 7. In this study, the first five components out of seventeen components contributed to 71% of the total variation, with an eigenvalue of more than one. Fifteen studied traits out of seventeen showed a positive contribution towards yield except for internode distance (−0.033) and hundred-seed weight (−0.015), expressing 20.01% of the total variability by PC2, where collectively both PC1 and PC2 acquainted 43.92% of the cumulative variation in the population.

#### 3.6.2. PCA Biplot

**Figure 3:**
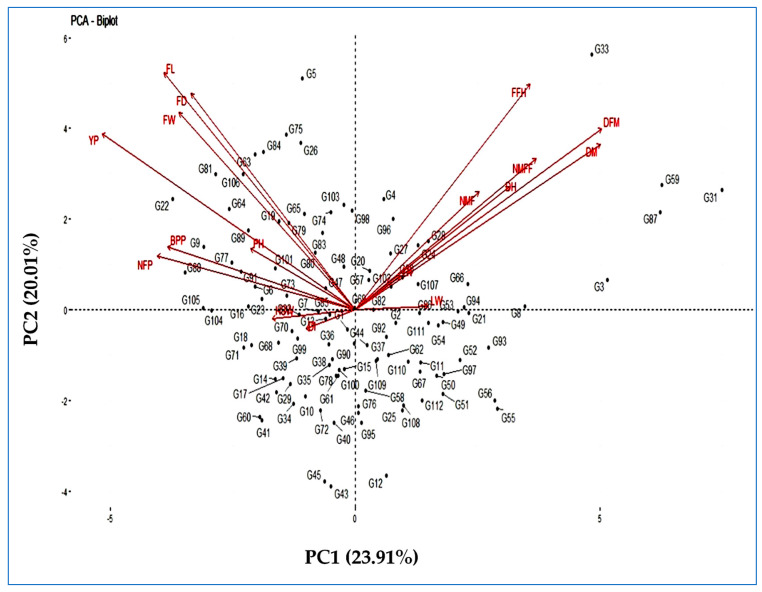
PCA plot of 103 cucumber landraces based upon the first two major components for morphological quantitative traits.

The landraces by traits biplot was constructed from a two-way matrix of 17 morphological quantitative traits and 103 cucumber landraces using the relative value of the trait (Figure 3). The biplot showed the trait profiles of the landraces, and the results indicated a correlation between traits with landraces. Again, it may be concluded that traits on opposite sides of the origin are negatively correlated and that traits near each other are positively correlated.

According to the results, variables such as days to a male flower, days to a female flower, the number of nodes at the first male flower, the number of nodes at the first female flower, days to the first fruit harvest, leaf length, leaf width, and days to a mature fruit harvest are close enough and form a small angle, representing a positive correlation between two variables. Similarly, plant height, primary branches plant^−1^, fruit length, fruit width, fruit weight, fruits plant^−1^, and yield plant^−1^ also form a small angle, representing a positive correlation. Therefore, during selection and/or choosing the parents for hybridisation in cucumbers for increasing yield and improving quality, a breeder must give greater attention to these characteristics. Moreover, traits regarding the origin at 90^0^ or more to each other are not correlated.

#### 3.6.3. Heatmap Analysis

**Figure 4:**
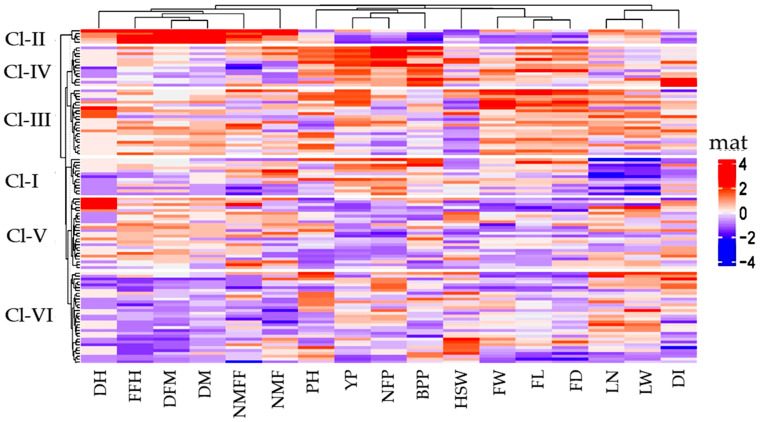
Heatmap showing the clustering pattern of 103 cucumber landraces with 17 morpho-physiological traits. Heatmap also displays the relationship matrix among cucumber landraces. PH = Vine length, BPP = Number of primary branches per plant, DI = Internode distance/internode length, DM = Days to a male flower, DFM = Days to a female flower, NMF = Number of nodes at the first appearance of a male flower, NMFF = Number of nodes at the first appearance of a male flower, LN = Leaf length, LW = Leaf width, FFH = First fruit harvest, FL = fruit length, FD = fruit width, FW = Fruit weight, NFP = Number of fruits per plant, DH = Days to a mature fruit harvest, YP = Yield per plant, and HSW = Hundred-seed weight.

A heatmap depicts a two-dimensional visual representation of data using colour changes from hues to a darker intensity, where the colours all represent different values. In the present study, a clustered heatmap was constructed to know the overall performance of the 17 observable traits among the 100 landraces with 3 checks (Figure 4).

A colour dissimilarity from dark to light indicates how the phenomenon is grouped or how it varies over space, thus making it easier to read. It also reveals the comparative form of highly abundant features against a background of mostly low-abundance features. Here, each column represents an individual characteristic, and each row is a measurement of that characteristic. Therefore, the heatmap analysis produced two dendrograms: one in the vertical direction, representing the landraces, and one in the horizontal direction, representing the traits that caused the diffusion. Based on the morphological properties of the landraces studied, six clusters emerged through hierarchical clustering. Another dendrogram showed four significant groups. Group one is associated with six traits (DH, FFH, DFM, DM, NMFF, and NMF). Group two is associated with four traits (VL, YP, NFP, and BPP). Group three consists of four traits (HSW, FL, FD, and FW). Group four is associated with three traits (LN, LW, and DI). The vertical dendrogram represents grouping among the landraces where landraces are divided into six clusters. Cluster members are given in Table 8. The hierarchical cluster analysis highlights the nature of relationships between some samples described by some type of descriptor. It classifies the landraces into different groups based on Euclidian distance. In the present study, based on genetic divergence, 103 diverse landraces of cucumbers were grouped into six clusters (Table 8).

The resultant six clusters showed phenotypic diversity, and the maximum number of landraces were accommodated in cluster VI (30), followed by cluster V (22), cluster III (22), cluster IV (14), cluster I (13), and cluster II (2). Genotype cluster mean values are shown in Table 9. Comparing cluster means with studied traits revealed considerable variation among different groups. Phenotypic divergence among the 103 landraces revealed that cluster III with landraces AC-14, AC-97, AC-471, AC-451, and RAI-209 was more divergent for improving average fruit weight, length, and width. On the other hand, cluster IV with landraces AC-201, TT-161, RAI-217, RAI-215, and TRMR-103 was more divergent for improving average vine length, internode length, and the number of primary branches plant^−1^, the number of fruits per plant, and yield per plant.

High colour consistency corresponding to characteristics FL, FD, and FW represents a relative pattern of a highly abundant feature of the values under cluster III. Similarly, a high colour consistency corresponding to characteristics VL, YP, NFP, and BPP represents a relative pattern of highly abundant feature of the values under cluster IV. Importantly, landraces AHI-116 and AHI-120, collected from saline areas Satkhira and Khulna, respectively, fell into clusters V and VI; in contrast, landraces RC-152 and AH-29, collected from drought areas Kurigram and Rajshahi, respectively, fell into cluster I and VI, exhibiting their diversity and importance for use in saline and drought-tolerant breeding programmes.

#### 3.6.4. Multi-Trait Index Based on Factor Analysis and Genotype–Ideotype Distance (MGIDI)

**Figure 5:**
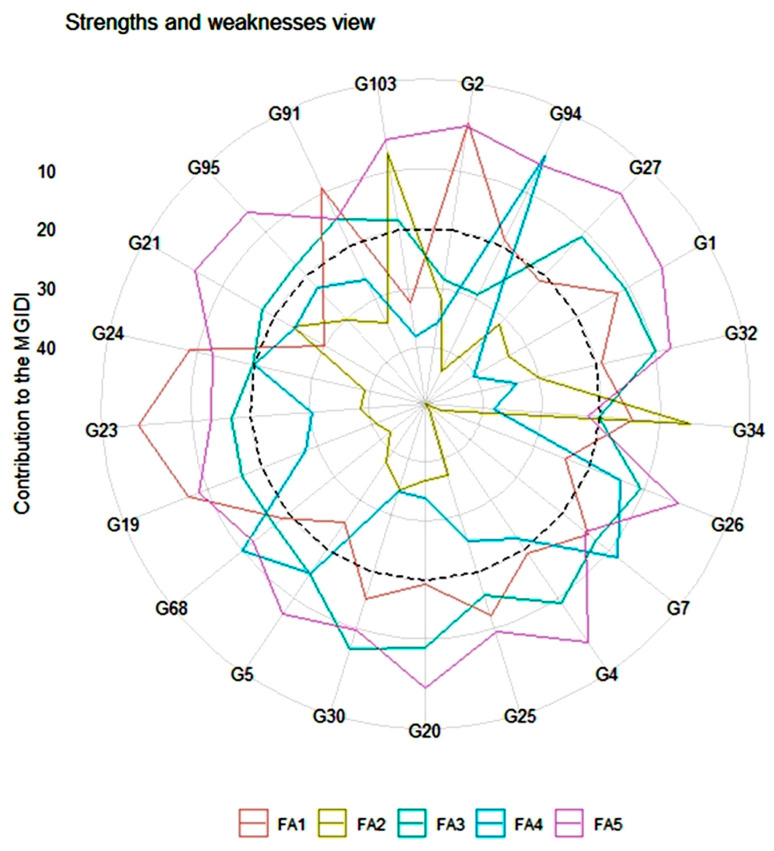
The strengths and weaknesses view of the selected landraces shown as the proportion of each factor on the computed MGIDI index.

Figure 5 shows the genotype ranking according to the MGIDI index for selecting the landraces with respect to considering all studied traits. The results show that a highly significant genotypic effect was noted for the traits of plant height (cm), primary branches plant^−1^, internode distance (cm), leaf length (cm), leaf width (cm), fruit length (cm), fruit width (cm), fruit weight (g), fruits plant^−1^, 100-seed weight (g), and yield plant^−1^ (kg) (Table 6).

**Figure 6:**
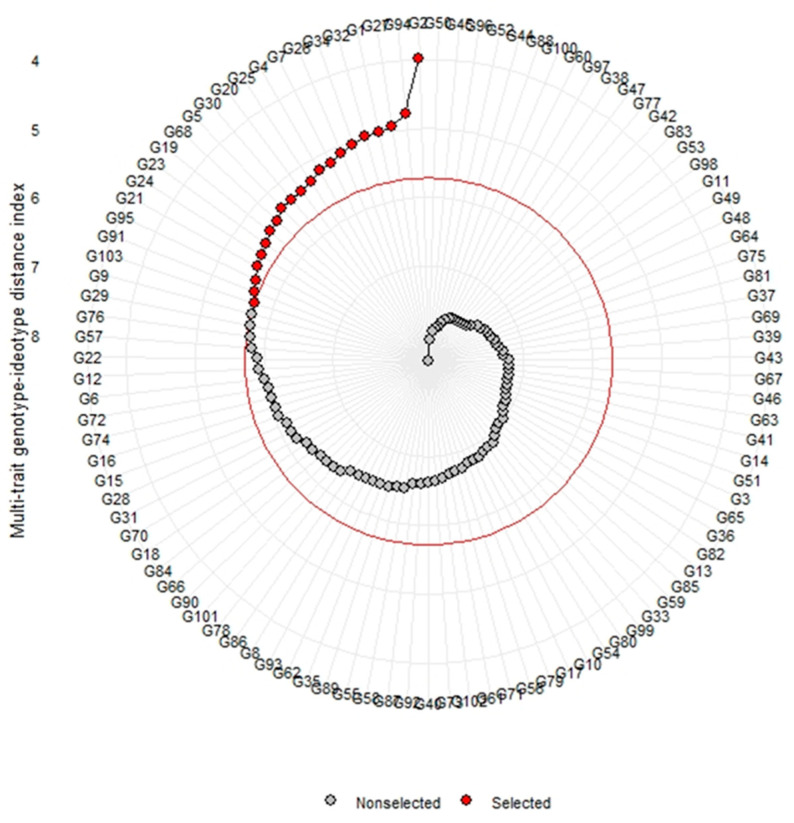
Genotype ranking in ascending order for the MGIDI index. The selected landraces based on this index are shown in red. As the proportion explained by a factor becomes smaller (closer to the external edge), the traits within that factor become closer to the ideotype. The dashed line shows the theoretical value if all the factors had contributed equally.

Broad-sense heritability (h^2^) was categorised as high for the above characteristics, such as plant height (94.41%), the number of primary branches per plant (84.17%), internode distance (92.07%), leaf length (82.51%), leaf width (79.66%), fruit length (87.61%), fruit width (73.94%), fruit weight (93.99%), the number of fruits per plant (84.88%), 100-seed weight (78.67%), and yield per plant (89.90%). All above-mentioned traits were evaluated with high heritability values along with the highest genetic advanced mean, which indicates that the selection gain of these traits is promising. According to the MGIDI index, landraces G1, G2, G4, G5, G7, G19, G20, G21, G23, G24, G25, G26, G27, G30, G32, G34, G68, G91, G94, G95, and G103 were selected (Figure 6). Figure 5 shows the strengths and weaknesses view of the selected landraces. The results of PCA show that the first five components with eigenvalues ≥ 1 accounted for 71.51% of the total variation among the traits. The strengths and weaknesses of the selected landraces show that the first factor (FA1) had the highest contribution for landraces G23, G24, G91, G2, and G19. The second factor (FA2) show the highest contribution for landrace G34. The third factor (FA3) indicates the highest contribution for landrace G30. The fourth factor (FA4) shows the highest contribution for landraces G68, G34, and G7. The fifth factor (FA5) represents the highest contribution for landraces G20, G5, G21, G95, G103, G27, G1, G32, G 26, G4, and G25 (Figure 5). The contributions of each factor to the MGIDI index were ranked from the most contributing factor (close to plot centre) to the least contributing factor (close to the plot edge).

## 4. Discussion

Understanding the nature and magnitude of the variability among the genetic stocks of the cucumber is of prime importance for breeders. In the present study, the phenotypic diversity of 103 cucumber landraces has been analyzed using several morphological qualitative and quantitative characteristics to quantify the yield potential of cucumber landraces, which can increase the effectiveness of landraces for breeding programmes. As a result of the degree of genetic variation, morphological characteristics are viewed as a critical initial step in characterising and identifying plant genetic resources [34].

### 4.1. Qualitative Traits

In our study, 17 qualitative characteristics have been studied, and among them, twelve qualitative traits were found to have significant variations, except for plant growth type, leaf blade shape, leaf pubescence density, flower colour, and sex type. Phenotypic variations were observed for almost all of the qualitative characteristics, including fruit shape, fruit skin colour, and flesh colour in *Cucumis melo* [35]. Variations were also displayed in 23 qualitative traits, viz., plant growth, stem shape, stem colour, stem and leaf pubescence density, flower colour, fruit shape, stem end fruit shape, blossom end fruit shape, fruit skin texture, fruit skin colour, etc., among 18 (9 *C. sativus* and 9 *C. melo*) accessions [36]. Fruit colour is an important trait which decides consumer preference. Our results demonstrate that the studied landraces had light green (65.05%), green (14.56%), dark green (3.88%), yellowish green (12.62%), greenish yellow (1.94%), blackish green (0.97%), and whitish green (0.97%) fruit skin colour. Wide variations in fruit colour have also been reported in [37,38]. Phenotypic diversity was the highest for the colour of leaves, size of leaves, skin colour of fruits, and shape of fruits [38]. Fruit shape is a significant quality factor for several domesticated plants [39,40]. Esteras et al. [41] found several kinds of cucumber fruits that were different in shape, colour, and size. In the current study, cucumber landraces were distributed into 4 types, viz., oblong (87 landraces), oval (5 landraces), ellipsoid (4 landraces), and ovate (7 landraces), based on fruit shape. Additionally, the lengths, diameters, and colours of fruit have been determined as important economic attributes [42,43]. Phenotypic variations in cucurbit crops are based upon fruit characteristics [44,45,46], which are useful for the differentiation of interrelated species [43]. The Shannon–Weaver diversity index analysis revealed the presence of genetic diversity based on qualitative traits among the accessions. The analysis of the genetic diversity index (H) for different qualitative characters ranged from 0 to 0.98, where 0 means no variation found for a particular characteristic among the studied collection. The overall average value of H for all characteristics was 0.41. In general, based on qualitative characteristics, the medium variation found among the studied genotype existed over a relatively limited geographical range. Al-Rawahi et al. [47] reported higher diversity (H = 0.68) for all morpho-agronomic characteristics of cucumber accessions in Oman. Such a type of variation in the diversity index was also found in African cucumbers [48] and Chinese cucumbers [49].

### 4.2. Descriptive Statistics of Quantitative Traits

Great variability was displayed among cucumber landraces for most of the traits. Vine length, as presented in Table 5, varied greatly among all cucumber landraces and ranged from 159.34 to 299.61 cm, indicating wide genetic diversity among the cucumber landraces. Our findings are similar to those of Abusaleha and Dutta [50] and Hossain et al. [51], who also studied vine length and found a wide variation. Significant variability was present in fruits per plant among all landraces, and the maximum number of fruits per plant was 9.75, whereas the minimum was 2.58. Hossain et al. [48] also reported that the number of fruits per plant varied significantly among different accessions. This variability may be due to genetics, the environment, and their interaction. In this study, fruit length showed great variation among all the landraces. The highest fruit length was recorded as 27.49 cm, and the lowest length was 10.8 cm, as shown in Table 5. These results are in agreement with those obtained by Sharma et al. [52]; Prasad and Singh [53]; and Munshi et al. [54], who also found significant differences in fruit length. The fruit width data presented in Table 5 revealed that different cucumber landraces exhibited differences, which ranged from 8.4 cm to 4.08 cm. Variations in fruit diameter were also reported by Soleimani and Ahmadikah [55] and Sharma et al. [52] in cucumbers. Our results show wide variations found in fruit weight, whereas the maximum fruit weight was recorded at 615.89 g, and minimum fruit weight was recorded at 143.06 g. Zhang et al. [36] stated that fruit weight displayed the biggest divergence among the nine quantitative traits in *Cucumis sativus.* A wide range was observed for the number of nodes bearing the first female flower (6.21–13.37), which determines the earliness of a variety (Table 5). Furthermore, from our study, noticeable variability was found in the number of primary branches per plant (3.17 to 7.04), internode distances (3.02 cm to 14.52 cm), and yield per plant (3.11 kg to 0.96 kg). Tremendous variability *in C. sativus* with respect to various horticultural traits was also reported by different authors [56,57,58,59,60,61,62]. Kanwar and Rana [63] observed that all the characteristics showed a wide range of values except for the days to the first picking of cucumbers, which was in line with our results.

The coefficient of variation (CV%) compares the relative amount of variability between crop plant traits [64]. In our experiment, a high CV% was obtained by the yield plant^−1^, the number of fruits plant^−1^, fruit weight, and fruit length. Moreover, quite high CV% was recorded for vine length and the number of primary branches per plant. These results imply that the number of branches, vine length, fruit weight plant^−1^, yield plant^−1^, and fruit length, in that order, had higher amounts of exploitable genetic variability among the studied cucumber attributes [65,66].

### 4.3. Genetic Variability Component

Understanding the magnitude of variability in crop species with respect to yield and yield-attributing traits in landraces is essential, since it provides the foundation for the selection of desirable types. In our study, the highest estimates (>20%) of the phenotypic coefficient of variation (PCV) and genotypic coefficient of variation (GCV) were observed for the internode distance, average fruit weight, number of fruits per plant, and yield per plant, indicating a wide range of variations (Table 6). Similar results for high GCV were also reported for fruit weight, branches per plant, and 100-seed weight [38]; for fruit weight, fruit yield per plant, and the number of fruits plant^−1^ [57]; and for fruit weight and fruit length [37]. A trait with a GCV value close to that of the PCV indicates the least possibility of environmental influence. Hence, selection based on phenotypic performance with a high GCV is more reliable.

The estimates of heritability (broad sense) varied from 24.33–94.41% for the different characteristics under study (Table 6). Characteristics, viz., vine length (94.41%), internode distance (92.07%), the number of primary branches plant^−1^ (84.17%), fruit weight (93.99%), fruit length (87.61%), number of fruits plant^−1^ (84.88), and yield plant^−1^ (89.9), were found as highly heritable traits. High heritability indicates less environmental influence in the observed variation [65]. Moderate heritability for the node number bearing the first female flower was observed in our results. This result is in line with those of [67] in cucumbers. Heritability accompanied by genetic advances is more useful for accessing more effective trait selection than heritability alone [54]. In the present study, high h^2^bs along with high genetic advance for plant traits such as vine length, the number of primary branches plant^−1^, internode distance, leaf length, leaf width, fruit length, fruit width, fruit weight, the number of fruits plant^−1^, and yield plant^−1^ suggested being more effective for selection. Due to the strong influence of the environment in expressing particular traits, selection based on traits with low to moderate heritability and genetic advance values is not wise, and in this case, heterosis breeding has the potential to improve those traits [68]. The same kind of outcome has also been reported in several studies for various traits in different crops [66,69,70].

### 4.4. Correlation Matrix

A correlation matrix is a statistical measurement used to determine the extent of associations between various plant characteristics, whether positive or negative, and thus, it helps to identify the characteristic for which selection can be imposed for improving the associated characteristics. In our study, fruit yield per plant exhibited a positive and significant correlation with the number of fruits plant^−1^, fruit length, fruit weight, fruit width, branches plant^−1^, and vine length, which indicated the importance of these traits in selection for yield. Vine length showed a positive correlation with branches plant^−1^, fruit length, fruit weight, and the number of fruits per plant. These results are also in accordance with the findings of [50,51,71,72]

### 4.5. Multivariate Analysis

The multivariate technique was used to determine associations between characteristics and to measure genotype genetic diversity. Although correlation studies help to determine a positive or negative association of an independent variable with the dependent variable (i.e., yield), in the case of a greater number of independent variables, their association becomes more complex.

#### 4.5.1. Principal Component Analysis

Principal Component Analysis (PCA) helps to identify the most relevant characteristics, explaining the maximum proportion of the genetic variation to the final yield. The results of PCA for the 17 traits of cucumber landraces that were evaluated indicate that the first five principal components had an eigenvalue of 1 and above, representing a cumulative variance of 71.51%. Among them, the first two components accounted for 43.92% of the cumulative variation in the population. Both PC components were positively and highly associated with the days to a male flower, days to a female flower, the number of nodes at the first female flower, the first fruit harvest, fruit length, fruit width, fruit weight, and the number of fruits per plant. Therefore, a breeder must use positive selection for those traits with a positive contribution towards yield. Similar findings were also reported by Olfati et al. [73] and Chikezie et al. [74] for cucumbers.

#### 4.5.2. PCA Biplot

The loading of different variables based on the first two principal components is represents in the biplot (Figure 3), indicating that variables such as days to a male flower, days to a female flower, the number of nodes at the first male flower, the number of nodes at the first female flower, days to the first fruit harvest, leaf length, leaf width, and days to a mature fruit harvest are close enough and form a small angle, representing a positive correlation between two variables. Similarly, plant height, the number of primary branches per plant, fruit length, fruit width, fruit weight, the number of fruits per plant, and yield per plant also form a small angle, representing a positive correlation. Similar results have been reported by many researchers, such as Zhang and Cui [75] and Kumar et al. [76] for cucumbers; Portis et al. [77] for peppers; and Koutsos et al. [78] for okra.

#### 4.5.3. Heatmap Analysis

The heatmap shows the highest and lowest values of each genotype in different colours from hues to a darker intensity against all the traits compared. The heatmap analysis depicts hierarchical clustering based on the morphological quantitative traits of the studied cucumber landraces, revealing six clusters and highlighting the nature of relationships between some samples, as described by some types of traits (Figure 5 and Table 9). Genetic divergence among the 103 landraces revealed that landraces under cluster III were more divergent for average fruit weight, fruit length, and fruit width. A high colour consistency in the heatmap corresponding to the characteristics FL, FD, and FW represents the relative pattern of a highly abundant feature of the values under cluster III. On the other hand, landraces under cluster IV were more divergent for average vine length (VL), internode length, the number of primary branches per plant (BPP), the number of fruits per plant (NFP), and yield per plant (YP). Similarly, a high colour consistency in the heatmap corresponding to the characteristics VL, YP, NFP, and BPP represent the relative pattern of the highly abundant feature of the values under cluster IV. Landraces were distributed into different clusters, indicating that geographical diversity may not necessarily be related to genetic diversity. A total of 28 diverse landraces of cucumbers were grouped into 6 clusters, which showed landraces from the same location in different clusters, indicating that geographical diversity may not necessarily be related to genetic diversity [79].

#### 4.5.4. Multi-Trait Index Based on Factor Analysis and Genotype–Ideotype Distance (MGIDI)

In the selection process, plant breeders usually assess multiple traits [80,81]. Plant breeders keep a plant ideotype in mind that represents selection for high-performance plants. An ideotype provides breeders with an ultimate target for selection, thereby consequently increasing plant performance through executing a stepwise trial–error method [82]. In breeding programmes, the ideotype-based selection process must be used, considering all the desirable relationships between the traits and the values for the traits. In our experiment, the cucumber landraces were ranked based on information on measured multiple traits (Figure 6). According to the MGIDI index, G1, G2, G4, G5, G7, G19, G20, G21, G23, G24, G25, G26, G27, G30, G32, G34, G68, G91, G94, G95, and G103 were selected, whereas G103 was very close to the cut point, which indicates that this genotype can exist with desirable features, and thus, the researcher should pay particular attention when assessing this genotype. The MGIDI index works as a powerful tool to develop better recommendation strategies, and different researchers use this index for different crops [83,84].

## 5. Conclusions

In the present study, a wide range of variability was found among landraces. Characteristics such as vine length, the number of primary branches per plant, internode distance, leaf length, leaf width, fruit length, fruit width, fruit weight, the number of fruits per plant, hundred-seed weight, and yield per plant showed high genetic variability coupled with high genetic advance. The landraces fell into six distinct clusters, among which clusters I, V, and VI contained landraces collected from saline and drought-prone areas. Using different multivariate analyses, i.e., using principal component analysis, a heatmap, and the MGIDI index, it could be concluded that selection is rewarding for vine length, the number of primary branches per plant, fruit length, fruit width, fruit weight, the number of fruits per plant, and yield per plant for bringing out improvements in cucumbers. A crossing programme between the landraces, collected from saline and drought-prone areas belonging to clusters I, V, and VI, along with the landraces of other clusters, could result in suitable cucumber varieties for forthcoming climate-changing conditions.

## Figures and Tables

**Table 1:** List of collected cucumber landraces from different parts of Bangladesh.

SL No.	Landrace Code	Collected Location	Geographical Location	SL No.	Landrace Code	Collected Location	Geographical Location
1	AH-19	Dinajpur	N-25°45.612′ E-88°40.734′	53	AC-294	Dhaka	N-23°55.826′ E-90°43.044′
2	AH-20	Dinajpur	N-25°45.612′ E-88°40.734′	54	AC-299	Dhaka	N-23°55.826′ E-90°04.344′
3	AH-29	Panchagarh	N-25°56.799′ E-88°47.088′	55	AC-305	Dhaka	N-23°55.826′ E-90°04.344′
4	AH-38	Bogra	N-24°66.099′ E-89°41.236′	56	AC-340	Gazipur	N-23°54.76′ E-90°30.473′
5	AH-63	Gazipur	N-23°59.503′ E-90°24.903′	57	AC-343	Gazipur	N-23°54.761′ E-90°30.473′
6	IAH-58	Rajshahi	N-24°28.012′E-88°19.506′	58	AC-379	Gazipur	N-24°02.070′ E-90°31.017′
7	IAH-74	Gazipur	N-24°05.562′ E-90°34.855′	59	AC-418	Dhaka	N-23°39.605′ E-90°21.566′
8	IAH-273	Bhola	N-22°13.352′ E-90°42.090′	60	AC-426	Narsingdi	N-24°05.735′ E-90°50.853′
9	IAH-274	Bhola	N-22°41.153′ E-90°38.751′	61	AC-451	Narsingdi	N-24°04.985′ E-90°53.282′
10	IAH-275	Patuakhali	N-21°84′ E-90°12′	62	AC-457	Narsingdi	N-24°04.985′ E-90°53.282′
11	IAH-323	Pirojpur	N-22°30.440′ E-89°57.502′	63	AC-471	Narsingdi	N-24°09.496′ E-90°48.603′
12	IAH-327	Jhalokathi	N-22°44.170′ E-90°11.124′	64	AC-495	Narsingdi	N-23°53.217′ E-90°44.899′
13	AMA-129	Mymensingh	N-24°34.085′ E-90°25.330′	65	AC-498	Narsingdi	N-23°53.217′ E-90°44.899′
14	AMA-204	Mymensingh	N-24°28.512′ E-90°28.133′	66	RAI-68	Chittagong	N-22°41.187′ E-91°46.506′
15	AMA-354	Sherpur	N-25°08.298′ E-89°52.938′	67	RAI-103	Chittagong	N-22°38.012′ E91°46.633′
16	AMA-406	Sherpur	N-25°16.482′ E-89°56.733′	68	RAI-106	Chittagong	N-22°38.012′ E-91°46.633′
17	AMA-413	Sherpur	N-25°16.887′ E-89°56.770′	69	RAI-116	Chittagong	N-22°38.012′ E-91°46.633′
18	AHI-05	Jhenaidah	N-23°26.474′ E-88°57.389′	70	RAI-117	Chittagong	N-22°38.012′ E-91°46.633′
19	AHI-15	Jhenaidah	N-23°26.474′ E-88°57.389′	71	RAI-122	Chittagong	N-22°19.049′ E-92°00.134′
20	AHI-22	Jhenaidah	N-23°27.049′ E-88°59.396′	72	RAI-127	Chittagong	N-22°19.049′ E-92°00.134′
21	AHI-26	Jhenaidah	N-23°27.049′ E-88°59.396′	73	RAI-137	Chittagong	N-22°19.049′ E-92°00.134′
22	AHI-33	Jhenaidah	N-23°27.021′ E-88°59.956′	74	RAI-149	Chittagong	N-22°18.182′ E-91°59.500′
23	AHI-34	Jhenaidah	N-23°26.474′ E-88°57.389′	75	RAI-209	Chittagong	N-22°09.666′ E-92°03.996′
24	AHI-35	Jhenaidah	N-23°26.474′ E-88°57.389′	76	RAI-215	Chittagong	N-22°30.020′ E-91°48.417′
25	AHI-41	Jhenaidah	N-23°24.427′ E-89°00.582′	77	RAI-217	Chittagong	N-22°30.020′ E-91°48.417′
26	AHI-48	Jhenaidah	N-23°18.190′ E-89°08.938′	78	RAI-245	Rangpur	N-25°49.007′ E-89°00.585′
27	AHI-49	Jhenaidah	N-23°18.190′ E-89°08.938′	79	RAI-253	Rangpur	N-25°49.007′ E-89^°^00.585′
28	AHI-70	Jhenaidah	N-23°18.190′ E-89°08.938′	80	RAI-255	Rangpur	N-25°49.007′ E-89°00.585′
29	AHI-72	Jhenaidah	N-23°18.190′ E-89°08.938′	81	RAI-265	Thakurgaon	N-26°01.711′ E-88°27.829′
30	AHI-78	Jessore	N-23°12.821′ E-89°11.123′	82	RC-07	Rangpur	N-25° 46.012′ E-89° 24.208′
31	AHI-89	Jessore	N-23°12.821′ E-89°11.123′	83	RC-31	Rangpur	N-25° 26.146′ E-89°21.038′
32	AHI-100	Jessore	N-23°12.828′ E-89°11.136′	84	RC-152	Kurigram	N-25°38.808 E-89°^41^.548′
33	AHI-113	Jessore	N-23°12.828′ E-89°11.136′	85	TR-2	Khagrachari	N-23°17.250′ E-91°54. 00′
34	AHI-116	Satkhira	N-22°45.030′ E-89°06.253′	86	TRMR-9	Cumilla	N-23°20.705′ E-91°12.087′
35	AHI-120	Khulna	N-22°47.320′E-89°27.445′	87	TRMR-10	Chandpur	N-23°04.012′ E-90°38.015′
36	AC-14	Dhaka	N-24°01.720′ E-90°12.480′	88	TRMR-85	Cumilla	N-23°27.755′ E-91°11.487′
37	AC-42	Dhaka	N-24°08.220′ E-90°13.530′	89	TRMR-103	Cumilla	N-23°22.520′ E-91°14.412′
38	AC-59	Tangail	N-24°24.501′ E-90°08.686′	90	TRMR-137	Cumilla	N-23°33.297′ E-91°07.555′
39	AC-74	Tangail	N-24°23.808′ E-90°11.435′	91	TT-06	Mymensing	N-24°34.807′ E-90°23.429′
40	AC-92	Tangail	N-24°19.197′ E-90°10.123′	92	TT-16	Mymensing	N-24°34.807′ E-90°23.429′
41	AC-97	Tangail	N-24°17.384′ E-90°05.30′	93	TT-94	Netrokona	N-24°49.817′ E-90°46.067′
42	AC-145	Narayanganj	N-23°48.140′ E-90°42.832′	94	TT-127	Netrokona	N-24°41.936′ E-90°46.452′
43	AC-149	Narayanganj	N-23°48.140′ E-90°42.832′	95	TT-161	Mymensing	N-24°42.155′ E-90°28.658′
44	AC-183	Narsingdi	N-24°01.020′ E-90°39.815′	96	ZS-01	Khagrachari	N-23°89.461′ E-91°84.209′
45	AC-184	Narsingdi	N-24°01.020′ E-90°39.815′	97	ZS-08	Khagrachari	N-23°91.341′ E-91°65.409′
46	AC-199	Narsingdi	N-23°58.752′ E-90°40.956′	98	ZS-17	Khagrachari	N-23°24.034′ E-92°05.069′
47	AC-201	Narsingdi	N-23°58.861′ E-90°41.315′	99	ZS-27	Khagrachari	N-23°24.034′ E-92°05.069′
48	AC-239	Manikganj	N-23°55.913′ E-90°00.788′	100	ZS-40	Khagrachari	N-22°59.055′ E-91°55.060′
49	AC-245	Manikganj	N-23°58.322′ E-90°02.207′	101	Shila	Lal teer Seed	N-23°59.503′ E-90°40.906′
50	AC-254	Manikganj	N-23°58.322′ E-90°02.207′	102	Baromashi	Metal Seed	N-23°59.503′ E-90°40.906′
51	AC-279	Dhaka	N-23°55.826′ E-90°04.344′	103	Baromashi	Lal Teer Seed	N-23°59.503′ E-90°40.906′
52	AC-281	Dhaka	N-23°55.826′ E-90°04.344′				

**Table 2:** Qualitative and quantitative descriptors and descriptor states for cucumbers.

Descriptor	Code	Descriptor State	Code	Descriptor State	Code	Descriptor State	Code	Descriptor State	Code	Descriptor State
Qualitative descriptors										
Plant growth type (at the vegetative stage)	1	Determinate	3	Indeterminate						
Plant growth habit (at the vegetative stage)	1	Viny	3	Intermediate	5	Prostate				
Stem colour (at the vegetative stage)		Light green		Green		Dark green				
Stem pubescence density (at vegetative stage)	1	Dense	2	Intermediate	3	Spares				
Leaf intensity of the green colour (at the vegetative stage)	1	Light	3	Medium	5	Dark				
Leaf shape (at vegetative stage)	1	Orbicular	2	Sagittate	3	Raniform	4	Cordate		
Leaf apex shape of terminal leaf lobe (at vegetative stage and fully developed leaf)	1	Obtuse	3	Rounded						
Leaf pubescence density (at vegetative stage and fully developed leaf)	1	Dense	2	Intermediate	3	Spares				
Flower colour (at fully developed flower)	1	White	2	Yellow						
Sex type (at fully developed flower)	0	Monoecious	1	Hermaphroditic	3	Androecious	5	Gynoecious		
Stem end fruit shape (at table maturity stage)	0	Necked	1	Acute	3	Obtuse				
Blossom end fruit shape (at the table maturity stage)	1	Flat	2	Deep raised	9	Other				
Fruit skin texture	0	Smooth	1	Wrinkle						
Fruit shape (at table maturity stage)	1	Oblong	2	Oval	3	Ellipsoid	4	Blossom end tapered	5	Ovate
Fruit skin colour (at table maturity stage)	1	Light green	2	Green	3	Dark green	4	Yellowish green	5	Greenish yellow
Fruit skin colour (at the mature harvest stage)	1	Brown	2	Yellow						
Seed colour	1	White	2	Cream						
Quantitative descriptors										
Vine length (cm)	The vine length was measured from the ground to the tip of the growing point with the help of a meter scale at the final harvest stage, and the average vine length per plant was calculated.									
Internode length (cm)	The distance between two adjacent nodes of the middle portion of the main stem was measured with the help of a scale and was expressed in centimetres, and the average internode length was calculated.									
Number of Branches/plants	Branches arising from the main stem were counted and noted at different intervals.									
Number of nodes on the main stem	The number of nodes on the main stem per plant was counted.									
Number of days to the male flower	The number of days taken from the date of sowing to the date of the first male flower appearing was recorded.									
Number of days to the female flower	The number of days taken from the date of sowing to the date of the first female flower appearing was recorded.									
Number of nodes at the first male flower	The number of nodes from ground level to the node at which the first male flower appeared was recorded.									
Number of nodes at the first female flower	The number of nodes from ground level to the node at which the first female flower appeared was recorded.									
Number of days to the first fruit harvest	The number of days from the date of sowing to the first picking of green fruit (table maturity) was recorded and expressed in days.									
Number of days to a mature fruit harvest	The number of days from the date of sowing to the first picking of mature fruit was recorded and expressed in days.									
Leaf length (cm)	The leaf lengths of 10 fully developed leaves were randomly taken, leaf lengths were measured, and the average was determined and expressed in cm.									
Leaf width (cm)	The leaf widths of 10 fully developed leaves were randomly taken, leaf width were measured, and the average was determined and expressed in cm.									
Fruit length (cm)	The lengths of individual fruits were measured using a scale of five randomly selected fruits at the edible stage.									
Fruit width (cm):	The widths of individual fruits were measured using a scale of five randomly selected fruits at the edible stage.									
Fruit weight (g):	The fruit weight was derived using the weights of five individual randomly selected fruits, and the average was determined.									
100-seed weight	The weight of 100 dried (12% moisture) seeds was determined.									
Number of fruits per plant	The total number of fruits harvested from each genotype was divided by the number of plants.									

**Table 3:** Qualitative descriptors and descriptor states of 103 cucumber landraces.

Descriptor	Descriptor State	Landraces (No.)	Observed Frequency	SWDI
Plant and leaf characteristics				
Plant growth type	Indeterminate	103	100	0
Plant growth habit	Viny	94	91.26	0.43647
	Intermediate	9	8.74	
Stem colour	Light green	4	3.88	0.66326
	Green	73	70.87	
	Dark green	26	25.24	
Stem pubescence density	Dense	61	59.22	0.61923
	Intermediate	42	40.78	
Leaf intensity of green colour	Light green	53	51.46	0.84547
	Medium green	41	39.08	
	Dark green	9	8.74	
Leaf blade shape	Orbicular	103	100	0
Leaf apex shape of the terminal lobe	Obtuse	72	69.90	0.44659
	Rounded	31	30.10	
Leaf pubescence density	Dense	103	100	0
Flower characteristics				
Flower colour	Yellow	103	100	0
Sex type	Monoecious	103	100	0
Fruit characteristics				
Stem end fruit shape	Necked = 1	1	0.97	0.60374
	Acute	30	29.13	
	Obtuse	72	69.90	
Blossom end fruit shape	Flat	100	97.08	0.12265
	Deep raised	3	2.92	
Fruit skin texture	Smooth	31	30.09	0.89317
	Wrinkled	72	69.90	
Fruit shape	Oblong	87	84.47	0.37973
	Oval	5	4.85	
	Ellipsoid	4	3.88	
	Ovate	7	6.80	
Fruit skin colour at the table maturity stage	Light green	67	65.05	0.35919
	Green	15	14.56	
	Dark green	4	3.88	
	Yellowish green	13	12.62	
	Greenish yellow	2	1.94	
	Blackish green	1	0.97	
	Whitish green	1	0.97	
Fruit skin colour at the mature harvest stage	Brown	72	69.90	0.56353
	Yellow	31	30.10	
Seed characteristics				
Seed colour	White	43	41.75	0.98582
	Cream	60	58.25	

**Table 4:** Analysis of variance of the tested quantitative traits in cucumber landraces.

Sources of Variation	Accession (G) with C (df-102)	Check (C) (df-2)	Accession vs. Check (1)	Accession (df-99)	Block (df-3)	Residuals (df-6)
PH	1574.41 **	2985.9 **	5339.58 **	1507.87 **	5.14 ns	84.28
BPP	0.58 **	0.77 *	11.13 **	0.47 *	0.13 ns	0.08
DI	4.39 **	8.6 **	8.41 **	4.27 **	0.37 ns	0.34
DM	23.81 *	464.08 **	405.68 **	11.06 ns	10.97 ns	6.31
DFM	26.7 ns	431.08 **	342.43 **	15.34 ns	4.75 ns	18.42
NMF	1.32 ns	3.06 ns	4.04 ns	1.26 ns	0.58 ns	0.95
NMFF	1.85 ns	3.94 ns	10.69 *	1.72 ns	0.24 ns	1.08
LN	5.14 *	4.37 ns	1.74 ns	5.19 *	0.56 ns	0.91
LW	7.57 *	0.38 ns	17.92 *	7.61 *	2.37 ns	1.55
FFH	16.1 ns	178.58 **	107.21 *	11.9 ns	10.33 ns	14.92
FL	13.33 **	20.03 **	22.03 *	13.11 **	1.1 ns	1.62
FD	0.91 *	1.16 ns	3.38 **	0.88 *	0.11 ns	0.23
FW	8176.14 **	16,532.02 **	5486.09 *	8034.5 **	413.8 ns	482.59
NFP	2.54 *	1.31 ns	15.3 **	2.43 *	0.14 ns	0.37
DH	18.18 *	34.08 **	0.00048 ns	18.04 *	4.31 ns	2.97
YP	0.41 **	0.65 **	2.08 **	0.39 **	0.01 ns	0.04
HSW	0.15 *	0.4 **	0.08 ns	0.14 *	0.18 ns	0.03

* 5% level of probability, ** 1% level of probability; ns = non-significant, PH = Vine length, BPP = number of primary branches plant, DI = Internode distance/internode length, DM = Days to a male flower, DFM = Days to a female flower, NMF = Number of nodes at the first appearance of a male flower, NMFF = Number of nodes at the first appearance of female flower, LN = Leaf length, LW = Leaf width, FFH = First fruit harvest, FL = fruit length, FD = fruit width, FW = Fruit weight, NFP = Number of fruits per plant, DH = Days to mature fruit harvest, YP = Yield per plant, and HSW = Hundred-seed weight.

**Table 5:** Descriptive statistics of quantitative traits of 103 cucumber landraces.

Trait	Max	Min	Mean	Std	CV
PH	299.61	159.34	221.00	38.38	17.37
BPP	7.04	3.17	4.60	0.77	16.66
DI	14.52	3.02	7.44	2.03	27.30
DM	57.5	30.42	39.11	4.25	10.87
DFM	65	39.08	47.59	4.34	9.12
LN	18.68	7.47	14.01	2.22	15.83
LW	24.42	8.62	17.38	2.85	16.40
NMF	9.54	3.54	5.78	1.08	18.74
NMFF	13.37	6.21	9.91	1.37	13.83
FFH	71.17	53.17	59.76	3.82	6.40
FL	27.49	10.8	17.53	3.60	20.53
FD	8.4	4.08	5.91	0.93	15.69
FW	615.89	143.06	298.60	86.17	28.86
NFP	9.75	2.58	5.41	1.55	28.71
DH	97.92	77.25	85.59	4.48	5.23
YP	3.11	0.96	1.93	0.62	31.88
HSW	3.81	1.82	2.68	0.51	19.03

The full names of the traits are given in the footnotes of Table 4.

**Table 6:** Estimations of statistical and genetic parameters of yields and their contributing traits.

Trait	PV	GV	GCV	GCV Cat.	PCV	PCV Cat.	h^2^ BS	h^2^ BS. Cat.	GA	GAM	GAM Cat.
PH	1507.87	1423.59	17.07	M	17.57	M	94.41	H	75.63	34.22	H
BPP	0.47	0.4	13.74	M	14.98	M	84.17	H	1.2	26.01	H
DM	11.06	4.75	5.57	L	8.5	L	42.97	M	2.95	7.54	L
NMF	1.26	0.31	9.57	L	19.4	M	24.33	L	0.56	9.74	L
NMFF	1.72	0.65	8.12	L	13.25	M	37.54	M	1.02	10.27	M
DI	4.27	3.93	26.62	H	27.74	H	92.07	H	3.92	52.7	H
LN	5.19	4.28	14.77	M	16.26	M	82.51	H	3.88	27.67	H
LW	7.61	6.06	14.17	M	15.87	M	79.66	H	4.53	26.08	H
FL	13.11	11.48	19.32	M	20.64	H	87.61	H	6.54	37.31	H
FD	0.88	0.65	13.7	M	15.93	M	73.94	H	1.43	24.3	H
FW	8034.5	7551.91	29.1	H	30.02	H	93.99	H	173.81	58.21	H
NFP	2.43	2.07	26.57	H	28.84	H	84.88	H	2.73	50.49	H
DH	18.04	15.07	4.54	L	4.96	L	83.53	H	7.32	8.55	L
HSW	0.14	0.11	12.5	M	14.09	M	78.67	H	0.61	22.87	H
YP	0.39	0.35	30.73	H	32.41	H	89.89	H	1.16	60.11	H

The full names of the traits are given in the footnotes of Table 4.

**Table 7:** The eigenvalues and contributions of different traits of cucumbers towards the major principal components.

Traits	PC1	PC2	PC3	PC4	PC5
VL	−0.151	0.103	−0.016	0.154	−0.510
BPP	−0.270	0.206	0.093	0.438	−0.114
DM	0.353	0.280	0.016	0.103	−0.086
DFM	0.354	0.307	0.007	0.180	−0.048
NMF	0.178	0.201	0.258	−0.163	−0.311
NMFF	0.260	−0.256	0.092	−0.089	−0.141
DI	−0.071	−0.033	−0.369	0.422	0.110
LN	0.065	0.057	−0.617	−0.181	−0.127
LW	0.104	0.006	−0.618	−0.166	−0.138
FFH	0.252	0.382	−0.013	0.170	−0.010
FL	−0.275	0.402	−0.007	−0.158	0.100
FD	−0.236	0.367	−0.015	−0.241	0.201
FW	−0.253	0.335	−0.022	−0.262	0.250
NFP	−0.285	0.292	−0.067	0.377	−0.256
DH	0.222	0.209	−0.093	0.204	0.220
HSW	−0.119	−0.015	0.027	−0.309	−0.570
YP	−0.364	0.299	−0.056	0.089	−0.037
Eigenvalue	3.962	3.209	2.152	1.543	1.297
Variability (%)	23.91	20.01	11.92	8.96	6.71
Cumulative variability (%)	23.91	43.92	55.84	64.80	71.51

The full names of the traits are given in the footnotes of Table 4.

**Table 8:** Distribution of 103 cucumber landraces in six clusters and landraces under each cluster.

Cluster (No.)	Landraces (No.)	Name of Genotype in Each Cluster
I	13	AC-279, AC-254, AHI-113, AHI-41, AHI-48, AHI-49, AMA 413, RAI-106, RAI-117, RAI-127, RAI-245, RC-152, TRMR-10
II	2	C3, C3, AH-38, C3, C3
III	22	AC-14, AC-145, AC-418, AC-42, AC-451, AC-59, AC-92, AC-97, AH-20, AC-457, AC-471, AC-495, AMA-204, RAI- 103, RAI-122, RAI-137, RAI-209, RAI-68, RC-31, TRMR- 137, TRMR-85, TT-06
IV	14	AC-183, AC-184, AC-201, AC-340, AC-498, AMA-129, RAI-116, RAI-215, RAI-217, RAI-253, TRMR- 9, TRMR-103, TT-16, TT-161
V	22	C2, AC-199, AC-426, C2, Iah-273, Iah-274, Iah-275, Iah-323, Iah-327, C2, AH-19, AH-63, AHI-120, RAI-149, C2, RAI-255, RAI-265, RC-07, TR-2, TT-94, ZS-01, ZS-08, ZS-17, ZS-27, ZS-40
VI	30	C1, AC-239, AC-245, AC-281, AC-294, AC-299, AC-343, AC-379, AC-74, C1, AH-29, AHI-05, AHI-100, AHI-116, AHI-15, AHI-22, AHI-26, AHI-33, AHI-34, AHI-35, AHI-70, AHI-72, AHI-78, AHI-89, AMA- 406, AMA-354, IAh-58, IAh-74, C1, AC-149, AC-305, C1, TT-127

**Table 9:** Cluster means for quantitative traits used in the classification of 103 cucumber landraces.

Cluster	PH	BPP	DM	DFM	NMF	NMFF	DI	LN	LW	FFH	FL	FD	FW	NFP	DH	HSW	YP
C-I	227.65	4.80	37.54	43.77	6.48	10.00	5.47	12.57	15.76	55.77	15.90	5.51	286.20	4.96	82.77	3.01	1.75
C-II	218.80	3.42	57.20	64.60	7.70	12.30	5.67	15.04	19.12	70.80	14.70	4.96	238.66	4.10	90.40	2.37	1.33
C-III	235.42	4.70	41.14	49.73	6.03	10.34	7.24	14.71	17.86	62.36	22.27	7.06	399.79	5.25	87.36	2.67	2.36
C-IV	247.06	5.52	38.14	47.43	5.35	9.64	9.00	12.57	15.82	60.93	18.89	6.08	318.92	7.64	84.57	2.68	2.72
C-V	198.03	4.13	39.52	49.48	6.25	10.54	7.09	13.70	17.01	60.76	15.43	5.58	249.17	4.16	86.04	2.74	1.30
C-VI	221.36	4.33	37.36	45.24	5.03	9.11	8.00	14.91	18.72	57.52	16.10	5.45	267.95	5.58	84.88	2.57	1.81

The full names of the traits are given in Table 4.

## Data Availability

Data are available upon request.
